# Computational insights in cell physiology

**DOI:** 10.3389/fsysb.2024.1335885

**Published:** 2024-03-13

**Authors:** Geneviève Dupont, Didier Gonze

**Affiliations:** Unité de Chronobiologie Théorique, Faculté des Sciences CP 231, Université Libre de Bruxelles (ULB), Bruxelles, Belgium

**Keywords:** systems biology, computational modeling, circadian rhythms, cell cycle, calcium signaling, entrainment, synchronization, cell differentiation

## Abstract

Physiological processes are governed by intricate networks of transcriptional and post-translational regulations. Inter-cellular interactions and signaling pathways further modulate the response of the cells to environmental conditions. Understanding the dynamics of these systems in healthy conditions and their alterations in pathologic situations requires a “systems” approach. Computational models allow to formalize and to simulate the dynamics of complex networks. Here, we briefly illustrate, through a few selected examples, how modeling helps to answer non-trivial questions regarding rhythmic phenomena, signaling and decision-making in cellular systems. These examples relate to cell differentiation, metabolic regulation, chronopharmacology and calcium dynamics.

## Introduction

Computational modeling is a powerful tool in physiology, allowing both to get mechanistic information from a given set of observations and to deepen our understanding of constantly evolving living systems (Keener and Sneyd, 2008). At the cellular level, models are built on molecular data and thus enable to closely simulate observed behaviors and make accurate predictions. However, despite the increasing use of computational approaches, models are still sometimes perceived by cell biologists as abstract and disconnected from concrete biological issues. A clear explanation, in biological terms, of the contributions from modelling to solving focused questions can help narrowing this gap. In this mini-review, we give four selected examples covering very diverse fields of cell physiology in which data-based computational modeling has played a significant role in providing insights into specific questions. We illustrate here how models can provide mechanistic explanations to sometimes unexpected cellular behaviors. Additionally, the predictive power of computational modelling can guide experimental investigation. In the following, the first example, which concerns cell differentiation during development, involves multistability. The three others, pertaining to the circadian clock, the cell cycle and calcium dynamics, involve oscillations. Multistability and oscillations both originate from non-linear interactions and multiple feedback loops (Goldbeter, 1996), which makes the use of a computational approach particularly useful.

## How does a population of (nearly) identical cells reproducibly give rise to two distinct cell populations during early embryonic development?

**FIGURE 1:**
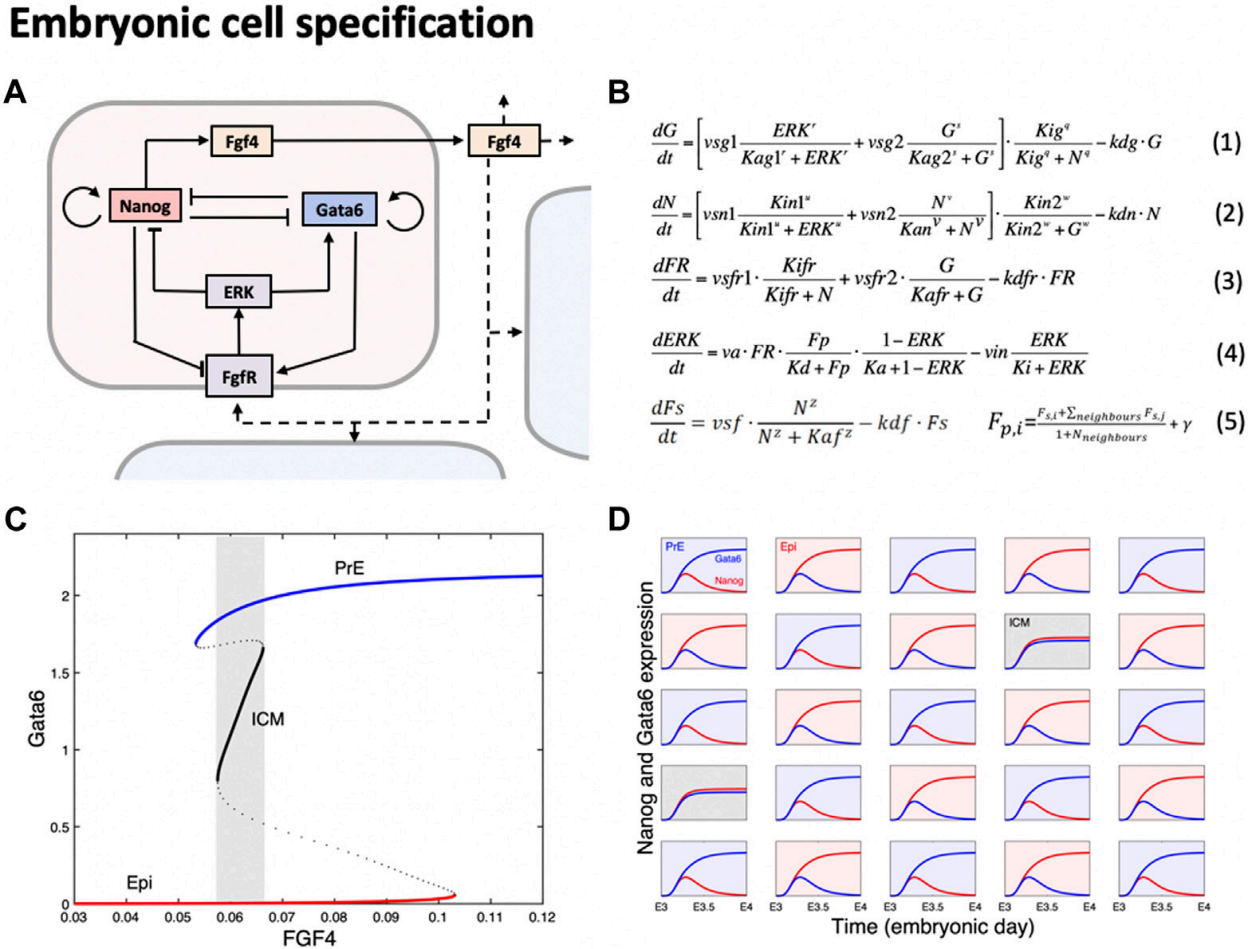
A GRN modulated by signaling, exhibiting tristability, underlies specification of cells of during early mouse development. Panel **(A)** shows a schematic representation of the core gene regulatory network in which NANOG and GATA6, the key transcription factors characterizing the Epi and PrE states, cross-inhibit and auto-activate. Cells secrete FGF4 at a rate that increases with the level of expression of Nanog. Because FGF4 activates the ERK pathway, this inter-cellular signaling allows for a crosstalk between neighboring cells. Panel **(B)** shows the equations corresponding to the GRN schematized in panel **(A)**. Ordinary differential equations give the time evolution of GATA6 (G), NANOG (N), FGF receptors (FR), the fraction of active ERK signaling (ERK) and secreted FGF4 (F_s_). When simulating a population of cells, evolution equations and parameter values are identical for each cell. The amount of FGF4 perceived by each cell (F_p,i_) is given by the local average of the FGF4 secreted by neighboring cells, modulated by a noise term (γ). Panel **(C)** shows a bifurcation diagram (De Mot et al., 2016) that indicates the steady states of a single cell GRN as a function of extracellular FGF4 (Fp). Equations 5 are thus not considered. Stable and unstable steady states are depicted by plain and dashed lines, respectively. For intermediate concentrations of FGF4 (grey area), the system has 3 stable steady states: one with high GATA6 and very low NANOG, corresponding to the PrE state (blue line), one with high NANOG and very low GATA6 corresponding to the Epi state (red line), and one where both NANOG and GATA6 are co-expressed at intermediate levels corresponding to the ICM state (black line). Panel **(D)** shows the outcome of a simulation of a population of 5 × 5 cells interacting through FGF4 signaling, giving rise to the characteristic salt-and-pepper pattern of Epi and PrE cells (shown with a red and blue background, respectively). As observed in the embryo, a small proportion of cells remain in the ICM state (shown with a grey background).

During development, cells from a population of common progenitors evolve towards different cell fates characterized by distinct levels of expression of specific transcription factors. This evolution is governed by gene regulatory networks (GRN) modulated by intercellular signaling. In the mammalian blastocyst-stage embryo, cells of the inner mass (ICM) differentiate into cells of the epiblast (Epi) or of the primitive endoderm (PrE) through a process that is both highly robust and noise-dependent. Indeed, the two populations of cells are generated in precise proportions and with a reproducible timing, but their spatial patterning is random as it exhibits a salt-and-pepper pattern (Chazaud et al., 2006). Models of the associated GRN revealed that ICM differentiation corresponds to a self-organized system relying on bi- or tri-stability (Bessonnard et al., 2014; Liebisch et al., 2020; Saiz et al., 2020; Raina et al., 2021; Stanoev et al., 2021). These models are all based on the cross-inhibition between the NANOG and GATA6 transcription factors that characterize the Epi and PrE states, respectively. Both factors are also regulated by the ERK pathway activated by extracellular FGF4. In the tri-stable scenario, cells initially coexpress NANOG and GATA6, as observed in cells that constitute the ICM. The time evolution of the FGF4-regulated GRN (Figure 1A) can be described by a system of 4 ordinary differential equations (Equations 1–4 in Figure 1B). For appropriate values of the parameters, the system exhibits tristability (Figure 1C) in a range of extracellular FGF4 concentrations (Fp). Initially, due to the presence of FGF4 of maternal origin, cells are in the intermediate ICM state. Evolution towards one of the differentiated states (De Mot et al., 2016) is then governed by the self-regulated secretion of FGF4 because cells secrete FGF4 at a rate that depends on their NANOG expression level (Equation 5 in Figure 1B). This allows cells to switch to the PrE state (when perceiving a high level of FGF4, activating the ERK pathway and leading to the synthesis of GATA6) or to the Epi state (when perceiving a low level of FGF4, deactivating the ERK pathway and allowing the synthesis of NANOG). When simulating a population of cells interacting with their neighbors through local secretion and perception of FGF4, a salt-and-pepper pattern of PrE and Epi cells, comparable to the mosaic arrangement observed *in vivo*, emerges (Figure 1D).

Validation of the proposed mechanism and related parameter values relied on the comparison with observations and on computational predictions. Simulations allowed to reproduce observations carried out under various experimental conditions including mutant embryos and embryos submitted to exogenous treatments that interfere with FGF4 signaling (De Mot et al., 2016; Tosenberger et al., 2017). Besides reproducing the outcome of these experiments, the model could predict that in average, Epi cells are specified earlier than PrE progenitors, which was validated experimentally (Bessonnard et al., 2014).

A key question relates to the possible sources of cell-to-cell heterogeneity initiating the specification of the ICM cells. As ICM cells are initially identical and evolve according to the same GRN, some asymmetry is required to trigger the separation of the developmental trajectories. Internal noise due to molecular fluctuations is not likely to play this role because once specified, cells are not observed to change fate, which would be the case in the presence of sufficiently large molecular noise (De Mot et al., 2016). Early models assumed the existence of extracellular heterogeneity in FGF4 concentration, reflected by the random and cell-specific value assigned to the parameter γ (Equation 5 in Figure 1B) that appears in the equation for the FGF4 concentration perceived by a cell (Tosenberger et al., 2017). However, observations in mouse embryos revealed the existence of heterogeneities in the levels of expression of some genes among cells even before FGF4 secretion (Allègre et al., 2022). This question was addressed by modelling as this allows to investigate the possible outcomes of simulated cell populations when varying the source of variability. Simulations predicted that the behavior of the model, in terms of dynamics of specification and final populations of cells of the different types, is preserved with respect to the source of variability (Robert et al., 2022). This property results from the existence of the intermediate ICM state that acts as a “buffer” to noise. As in toggle-switch based models that do not consider the existence of a third, intermediate stable steady state, signaling through FGF4 appears as a key factor to maintain reproducible proportions of the different cell types (Mathew et al., 2019; Saiz et al., 2020; Raina et al., 2021).

More elaborated studies, based on a statistical analysis of experimental data related to the levels of expression of the various genes at early developmental stages, are thus required to pinpoint the mechanism that drives the initial differentiation step from a pool of common progenitors and robustly enables the emergence of balanced proportions of the two cell types.

## Why can ill-timed feeding lead to altered metabolic regulation and to diseases?

Many physiological processes are regulated by the circadian clock and consequently follow a 24 h rhythmic pattern (Patke et al., 2020). Besides the sleep-wake cycle, hormone release, feeding behavior, nutrient absorption and digestion, and glucose homeostasis change over the course of the day. The clock has evolved to organize physiological processes in time in order to optimize energy consumption and to anticipate predictable daily environmental changes. Perturbing these conditions, for example through jet lag or by ill-timed feeding, may lead to a dysregulation of this timing system. This is manifested by alteration of the clock genes expression and by metabolic syndromes. Feeding at the “wrong” time or irregular meal timing can indeed disrupt the ability of the organism to regulate blood glucose levels effectively. If repeated, these perturbations can cause fluctuations in blood sugar levels, potentially leading to insulin resistance and to an increased risk of diabetes (Tahara and Shibata, 2016; Stenvers et al., 2019).

Glucose homeostasis relies on insulin secretion by pancreatic beta cells. The clock of beta cells regulates the rhythmic transcription of genes involved in glucose-stimulated insulin secretion (Stenvers et al., 2019). This peripheral clock itself receives signals from the brain pacemaker clock and from food uptake. Once these two zeitgebers are not aligned (i.e. desynchronized), the amplitude and phase of core clock genes (including *Per, Bmal1*, and *RevErbα*) are altered. In turn, insulin secretion is not adjusted to the need, which leads to hypoinsulinemia and hyperglycemia (Mukherji et al., 2015a,b).

**FIGURE 2:**
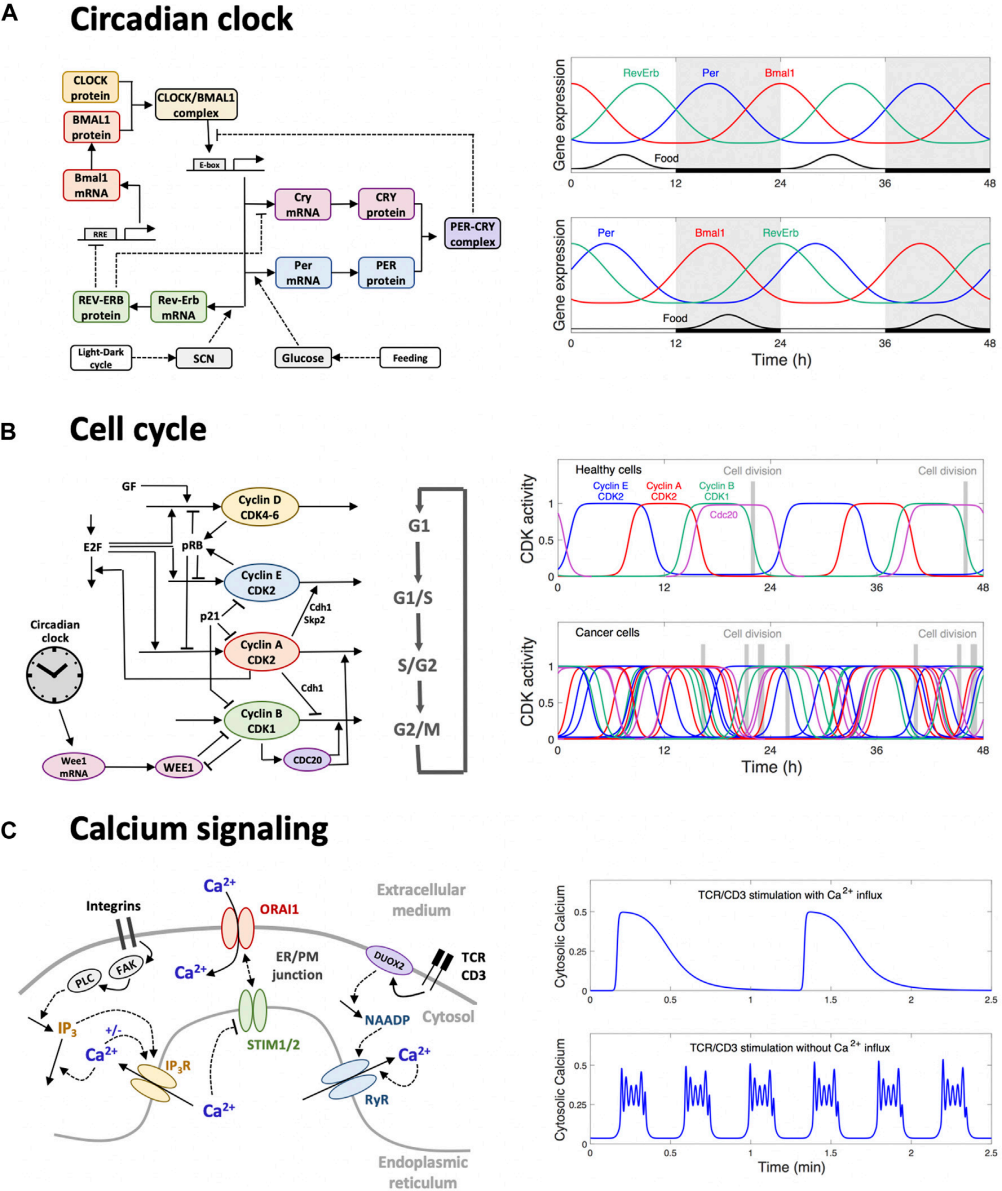
Three examples of oscillatory behaviors successfully addressed by computational modeling. **(A)** Circadian oscillations originate from a gene regulatory network involving interlocked transcriptional feedback loops (left panel). In peripheral tissues, such as the liver or the pancreas, the circadian oscillators receive inputs from the pacemaker clock located in the brain and responsive to the light-dark cycle, and from nutrient uptake. Wrong-time feeding disrupts the amplitude and phase relationship between the expression profiles of clock genes (right panel), which results in altered clock-controlled outputs such as insulin secretion. In turn, these dysregulations lead to physiological disorders, including hyperglycemia. **(B)** The progression of the cell into the successive phases of the cell division cycle is governed by a CDK/cyclin network (left panel). Healthy cells are well entrained by the circadian clock such that cells tend to divide at a certain time of the day (top right panel). In cancer cells, the circadian control is often altered. When the coupling strength is weak, many cells are not entrained and thus divide at any time of the day (bottom right panel). This feature can be exploited to optimize the administration time of anti-cancer drugs (chronotherapy). **(C)** Elements of the Ca^2+^ signaling toolkit in T cells. Adhesion stimulates phospholipase C (PLC) through the activation of the focal-adhesion kinases (FAK). The rise in IP_3_ that follows PLC activation provokes the release of Ca^2+^ from the endoplasmic reticulum (ER). The resulting local depletion in ER Ca^2+^ activates Ca^2+^ entry into the cytoplasm at the level of the ER/PM junction. This localized Ca^2+^ entry creates Ca^2+^ microdomains, which are highly localized in time and space. Early after TCR/CD3 stimulation, the nicotinic acid adenine dinucleotide phosphate (NAADP) synthesized by the Dual oxidase 2 (DUOX2) enzyme activates Ca^2+^ release via the ryanodine receptor (RyR), which also creates Ca^2+^ microdomains with very similar characteristics. Later after stimulation, IP_3_ is further increased by TCR/CD3 stimulation (not shown in the scheme) leading to global Ca^2+^ signaling in the form of Ca^2+^ oscillations. In physiological situations including Ca^2+^ influx, oscillations are long-lasting, with a low frequency (top right panel), inducing the translocation of nuclear factor of activated T-cells (NFAT) and the activation of the immune response. These oscillations are based on the STIM/ORAI mechanism. In the absence of influx, oscillations are faster and of the bursting type (bottom right panel), which reveals the interaction of other potentially active oscillatory mechanisms.

To better understand the interplay between the genetic dysregulation of the pancreatic circadian clock and its consequence for glucose homeostasis, a mathematical model was proposed by Woller and Gonze (2018). The model accounts for the circadian gene network (Figure 2A), as well as the clock-mediated dynamics of the glucose-insulin circuit, and takes the form of a set of ordinary differential equations governing the time evolution of gene expression and the concentration of glucose and insulin. The Hill-based functions used to describe the transcription rate as a function of the transcription factors provide the nonlinearity required to generate oscillations. Kinetic parameters are estimated by fitting the dynamics of gene expression and of glucose/insulin profiles to experimental time series obtained in mice (Mukherji et al., 2015a,b). The model is then used to simulate the system in pathological conditions, namely when food and light cues are misaligned (reflecting ill-time feeding). These simulations suggest that peripheral clocks may not completely uncouple from the central clock when food intake is inverted but rather lead to a differential phase shift in clock gene expression. Indeed, not all clock genes are phase-shifted to the same extent (Figure 2A). The simulations further show how this differential phase shift leads to a reduction of insulin secretion and to an increase of glucose, as well as to a loss of food anticipation. This study thus illustrates how computational modeling can complement experimental observations to raise hypotheses on the interplay between the genetic circadian oscillator and the onset of clock-related metabolic disorders (Woller and Gonze, 2021).

## At what time should anti-cancer drugs be administrated?

The progression of a cell through the different phases of the cell division cycle is governed by a network of CDK/cyclin complexes, which are sequentially activated through reversible phosphorylation/dephosphorylation (Figure 2B). The circadian clock controls this regulatory network at the molecular level, namely through the transcriptional regulation of several cell cycle components (Feillet et al., 2015; Farshadi et al., 2020). As a consequence, cells tend to replicate their DNA and to enter mitosis at specific times of the day. In cancer cells, the circadian clock is often impaired and sometimes decoupled from the cell cycle (Feillet et al., 2015). This may result in less synchronized cell division of cancer cells, which tend to divide independently of the time of the day (Figure 2B). This feature explains, at least in part, why cell-phase specific drugs exhibit differential effects depending on the time at which they are administrated. Moreover, the pharmacokinetic and pharmacodynamic parameters of drugs may also follow a circadian pattern. Chronopharmacology aims at understanding and exploiting these features in order to develop drug administration protocols which minimize their toxicity and maximize their efficacy (Ballesta et al., 2017; Amiama-Roig et al., 2022).

The cell cycle and the circadian clock can be seen as a system of oscillators mutually coupled through several molecular mechanisms. Understanding the dynamics resulting from such a complex system is not straightforward but is required to develop chronopharmacological treatments on a rational basis. Computational models contribute to determine the conditions under which the circadian clock effectively entrains the cell cycle as well as the causes of loss of synchronization.

Gérard and Goldbeter (2012) used detailed computational models for the two oscillators to show, through numerical simulations, that the cell cycle can acquire a period of 24 h once coupled to the circadian clock when its autonomous period is around 24 h. In contrast, the CDK/cyclin oscillator exhibits complex dynamics including chaos when its autonomous period is out of the entrainment range or when the coupling strength is too low. In these conditions cell division occurs independently of the circadian clock. The simulations also indicate that the combination of multiple modes of coupling does not necessarily facilitate entrainment of the cell cycle, suggesting that impairing some coupling mechanisms may not necessarily lead to a loss of synchronization. Leung et al. (2023) investigated the circadian forcing of the cell cycle at the population level in presence of variability on kinetic parameters. Assuming that the coupling strength is reduced in cancer cells, simulations predict that a population of cancer cells will encompass a certain fraction of non-entrained cells, which then may be targeted by a drug administrated at a time at which healthy cells are not sensitive to it. A characterization of the variability and of the coupling strength would then be needed to make such models quantitative so that their predictions may guide further experimental investigations.

In the above studies, the authors focused on the regulation of the cell cycle by the circadian clock. The reverse coupling was taken into account by Yan and Goldbeter (2019) and by Almeida et al. (2020). Yan and Goldbeter (2019) reported that including the effect of the cell cycle on the circadian clock leads to an increased robustness, to a reduction of complex oscillations, and to the emergence of multi-rhythmicity (coexistence of different coupling modes). Almeida et al. (2020) investigated the role of growth factors (GF) and dexamethasone (Dex) on the entrainment pattern of the cell cycle once coupled to the circadian clock. The authors predict that addition of GF, which stimulates the synthesis of the mitosis-promoting factor, leads to a decrease of the period of the coupled system, while increasing the level of Dex may drive the system from a 1:1 regime (one cell division per circadian cycle) to a 3:2 regime (2 cell divisions every 3 circadian cycles). The authors also simulated the effect of a pulse of Dex and observed a time-of-the-day-dependent response, i.e. a shift to a 3:2 synchronization mode. The results of these two studies may explain the emergence of distinct groups of cells in unsynchronized cell population, as observed in experiments. Finding ways to control the synchronization mode and the entrainment phase is critical in chronotherapy because this will dictate the optimal time of drug administration. Mathematical models allowing to test various situations and to characterize complex behavior for large parameter ranges may be helpful in this task.

## How does Ca^2+^ signaling activate the immune response in T cells?

Changes in Ca^2+^ concentrations are widely used to convey the information from outside to inside the cell, which led to qualify this ion as a “universal second messenger”. Strikingly, the temporal and spatial characteristics of these changes in Ca^2+^ concentration in the cell cytoplasm play a crucial role in the specificity of the physiological responses of the cell to Ca^2+^ signaling. These take most of the time the form of oscillations with various amplitudes, shapes and frequencies (Berridge, 1997). Highly localized and short durations Ca^2+^ increases, creating Ca^2+^ microdomains, have also been observed and reported to be associated with specific functions (Berridge, 2006). These events rely on a rather limited number of Ca^2+^ transporters and channels allowing for Ca^2+^ transfers between the cytoplasm and the other cell compartments, such as the extracellular medium, the endoplasmic reticulum or the mitochondria. Based on a kinetic description of these Ca^2+^-transporting elements arranged in a cell type- and stimulus-specific manner (Berridge et al., 2000; Parys and Bultynck, 2023), computational modeling is much used to help deciphering the molecular mechanisms underlying each specific type of Ca^2+^ response (Dupont et al., 2016).

Ca^2+^ signaling plays an essential role in T cell activation, which is key to initiate an adaptative immune response (Trebak and Kinet, 2019; Wang et al., 2020). The main elements of the T cell “calcium toolkit” are schematized in Figure 2C. As a first step in the transition from a quiescent to a fully activated state, Ca^2+^ microdomains (Wolf and Guse, 2017; Gil Montaya et al., 2023) are created in the junctions between the plasma membrane (PM) and the endoplasmic reticulum (ER). Such a small scale Ca^2+^ signaling sensitizes T cells allowing them to respond efficiently once fully activated (Weiß et al., 2023). The ER-PM junctions have a depth of approximately 15 nm and an extension of ∼200 nm (Hogan, 2015). Microdomain formation is dependent on Ca^2+^ entry from the extracellular medium through store-operated Ca^2+^ entry (SOCE). The latter mechanism relies on the stromal interaction molecules (STIM1 and/or STIM2) that are ER Ca^2+^ sensors regulating the activity of the Orai1 PM Ca^2+^ channels that allow Ca^2+^ entry from the extracellular medium into the cell. Thus, upon local depletion of the ER Ca^2+^ store, Ca^2+^ dissociates from STIM, which allows recruitment of Orai1 to the junction and their gating. Although current techniques in microscopy allow the observation of Ca^2+^ microdomains in the ER-PM junctions, they fail to fully address the mechanism of their formation that involves Ca^2+^ changes in the portion of the ER apposed to the junction. In particular, models are required to predict the number and spatial arrangement of the channels inside the junction (McIvor et al., 2018; Gil et al., 2021; Gil et al., 2022). Spatially resolved models using Comsol Multiphysics (COMSOL Multiphysics^®^ v. 5.4. www.comsol.com) can simulate the Ca^2+^ fluxes at the ER-PM junction and accurately reproduce observations based on high resolution microscopy. In this framework, partial differential equations describe changes in Ca^2+^ concentrations due to diffusion in the different compartments while Ca^2+^ channels/pumps enter in the model through the appropriate boundary conditions. Taking into account measured values for the rates of Ca^2+^ fluxes, modeling shows that Ca^2+^ microdomains corresponding to those seen in experiments can be simulated when considering that cell adhesion activates FAK (focal adhesion kinase), stimulating the IP_3_-synthetizing enzyme PLC (phospholipase C). Quantitative agreement between observations and simulations is best achieved when assuming that IP_3_ increases are such that they provoke the opening of 3–6 IP_3_ receptors/Ca^2+^ channels (Gil et al., 2021). The resulting local depletion of the ER activates the opening of a corresponding number of ORAI1 channels that create the microdomain. Interestingly, the model predicts that the microdomains created in the first ∼15 s following T cell receptor activation, although similar in spatial and temporal extents, are in contrast created by the openings of ∼7 large conductance ryanodine receptors (RyR) following the stimulus-induced increase in the local concentration of NAADP (Gil et al., 2022).

Later after T cell receptor activation, Ca^2+^ signaling takes the widespread appearance of repetitive spikes that, by stimulating the activity of the Ca^2+^-sensitive calcineurin phosphatase, promote the translocation of the nuclear factors of activated T cells (NFAT) to the nucleus. There, it promotes cellular responses such as cytokine production, proliferation, metabolism or differentiation (Trebak and Kinet, 2019; Wang et al., 2020). Because NFAT translocation is highly dependent on the temporal pattern of cytosolic Ca^2+^ changes (Fisher et al., 2006; Cooling et al., 2009), deciphering the molecular mechanism driving Ca^2+^ oscillations is key to understand–and possibly control–T cell immune responses. This mechanism differs from that occurring in most cell types and remained unclear until recently, largely because apparent conflicting results about the respective contributions of internal and extracellular Ca^2+^ stores. A recent study, involving both observations and modelling based on ordinary differential equations, allowed to clarify this question (Benson et al., 2023).

Two qualitatively different types of oscillations can be observed in this cell type. In conditions allowing Ca^2+^ influx from the external medium, low period (∼1 min), sinusoidal Ca^2+^ oscillations can be initiated by any means associated with a decrease of ER Ca^2+^. This includes increases in IP_3_ or in the RyR agonist NAADP, which are both synthesized in response to stimulation (Kurneth et al., 2003). In contrast, when Ca^2+^ influx is hindered, fast oscillations (∼10s) of the bursting type are observed following an increase in IP_3_ concentration. Importantly, in natural conditions, TCR stimulation activates IP_3_ synthesis and the extracellular Ca^2+^ concentration is elevated. From a theoretical point of view, slow and smooth oscillations are known to often result from a negative feedback mechanism involving a time delay (Goldbeter, 1996). Here, Ca^2+^ entry is inhibited by ER refilling, which is a slow process that involves the dissociation of STIM-ORAI complexes and the translocation of STM proteins (Croisier et al., 2013). On the other hand, oscillations of the bursting type generally involve the interplay between two oscillators (Borghans et al., 1997). This led Benson et al. (2023) to propose that fast Ca^2+^ oscillations in T cells result from a modulation of the IP_3_ concentration associated with an autocatalytic feedback due to Ca^2+^-induced Ca^2+^ release. The distinction between two oscillatory mechanisms is crucial given that STIM-ORAI based Ca^2+^ oscillations activate NFAT, while this step that is crucial for T cell activation is not induced in the absence of Ca^2+^ influx (Trebak and Kinet, 2019; Benson et al., 2023).

## Conclusion

From circadian clocks to development, the dynamics of cellular systems is dictated by regulated gene expression, post-translational modifications and signaling. Operating at the level of single cells, these molecular mechanisms give rise to well-organized and robust behaviors, while keeping the ability to adapt their conduct to changing conditions or to react acutely to a particular stimulus. Because of the ever-increasing accumulation of quantitative data and the power of technical methods for their analysis, computational modeling represents a unique way to decipher the molecular mechanisms that dictate cell’s survival and functioning. There is no doubt that the synergy between experimental observations and computational approaches will keep playing an increasing role in cell physiology.
